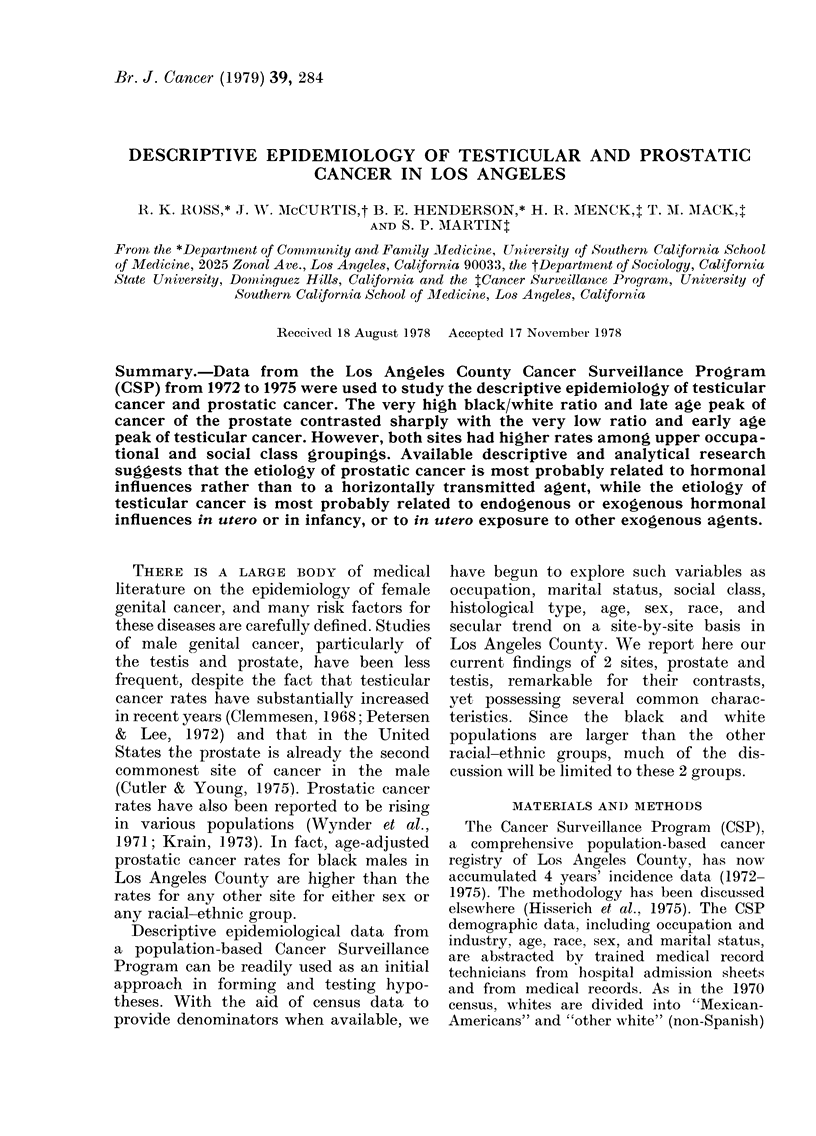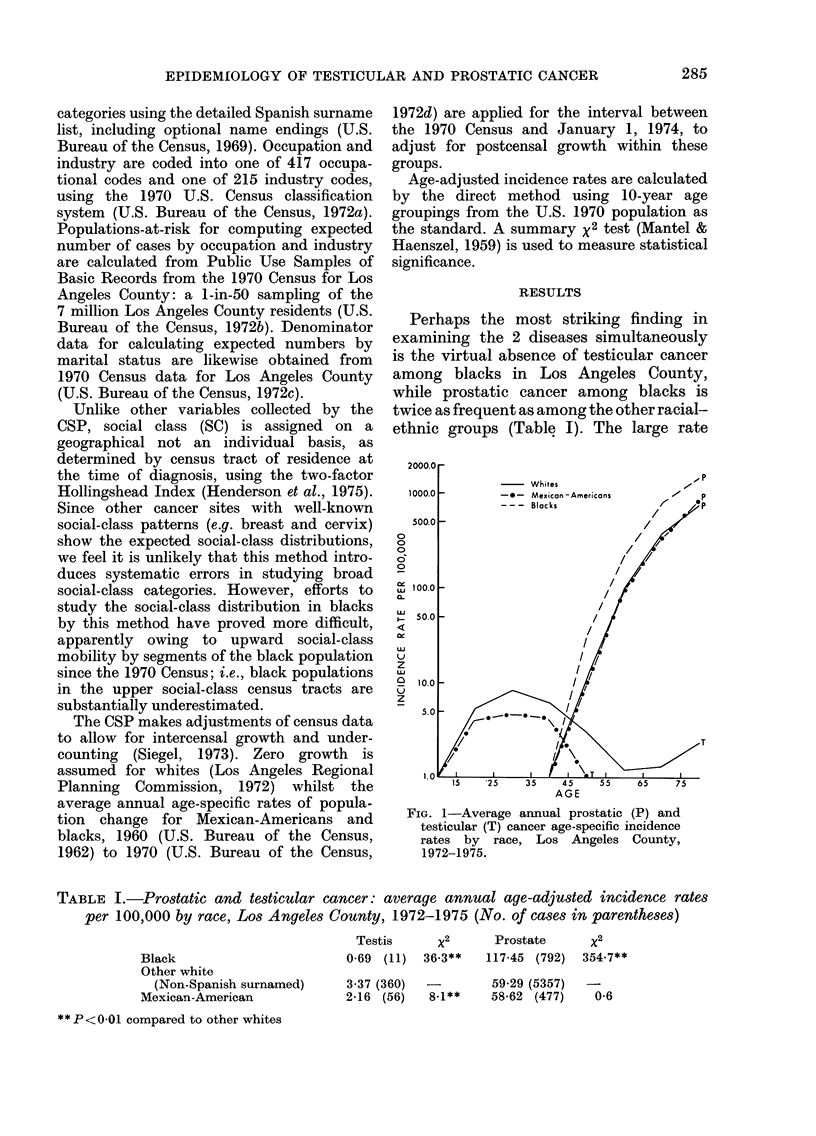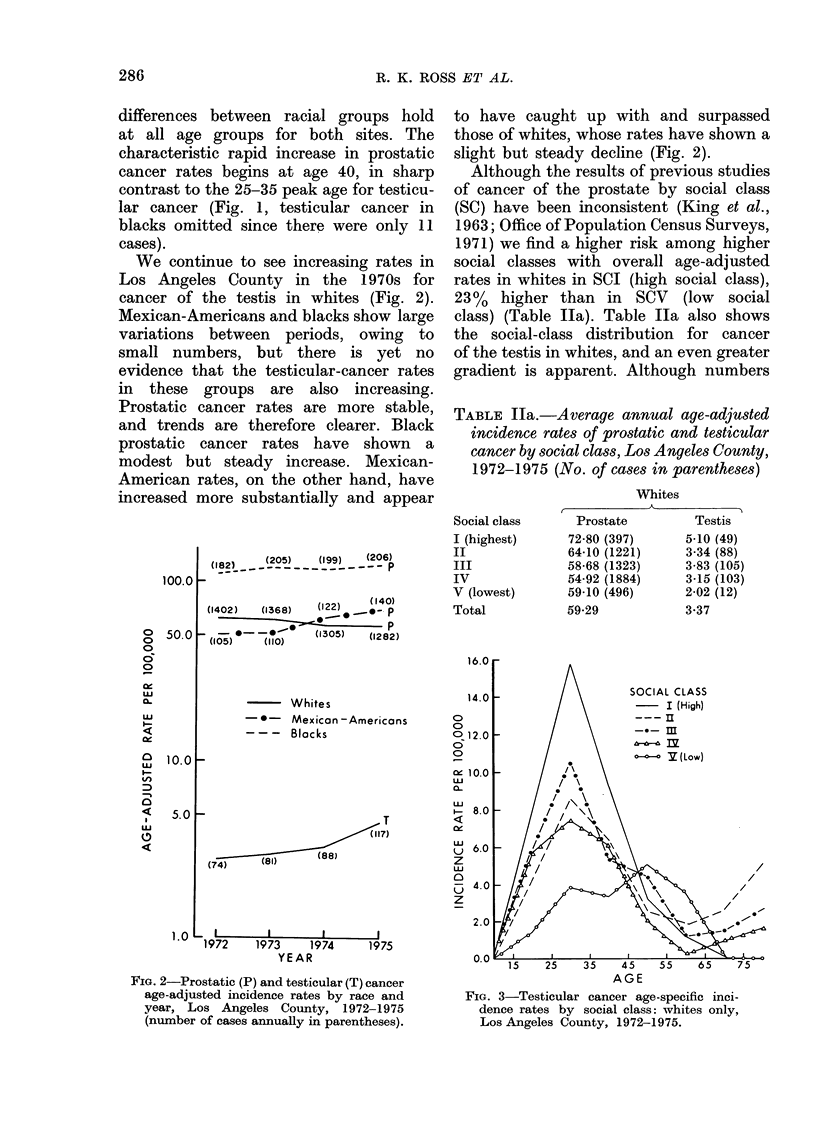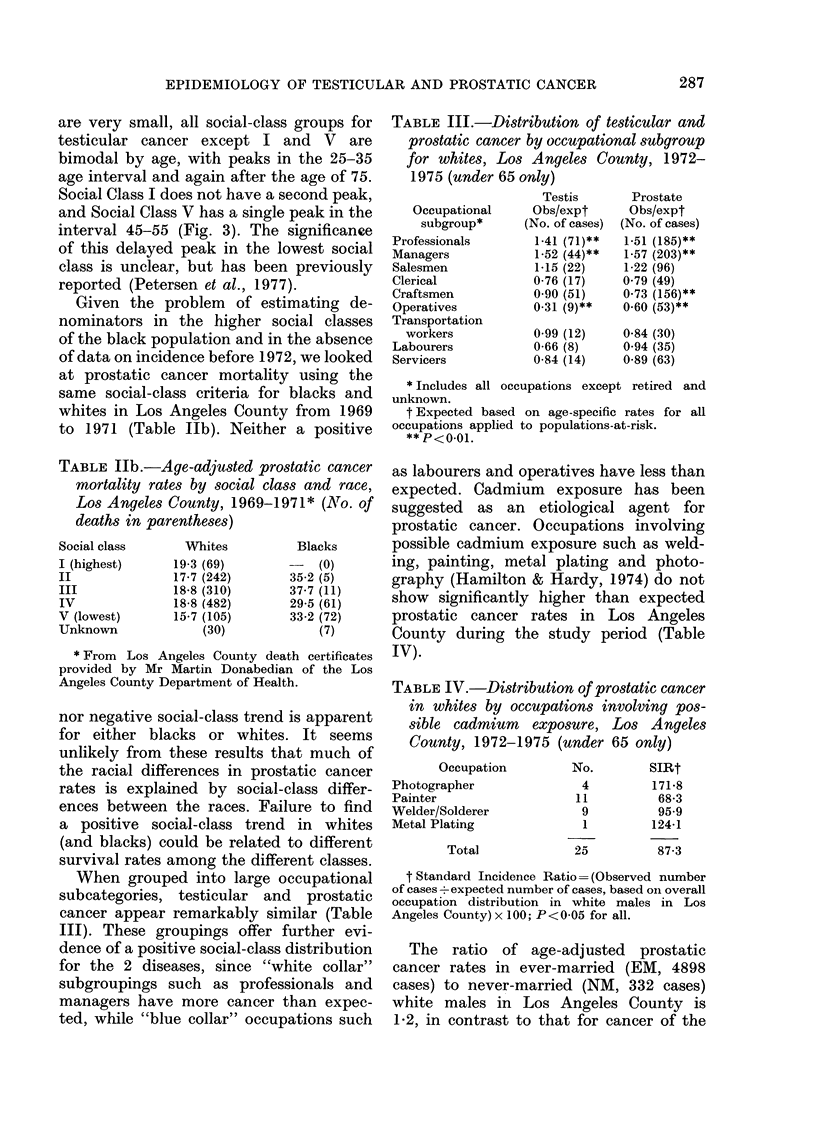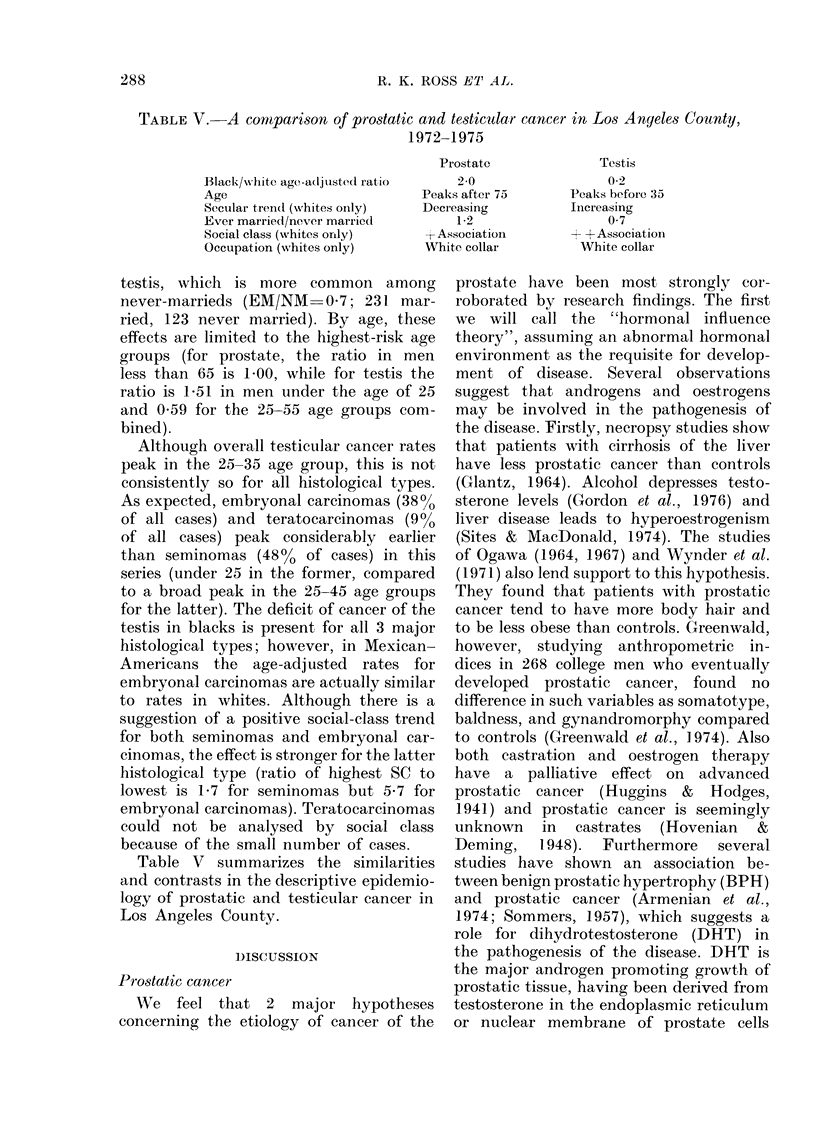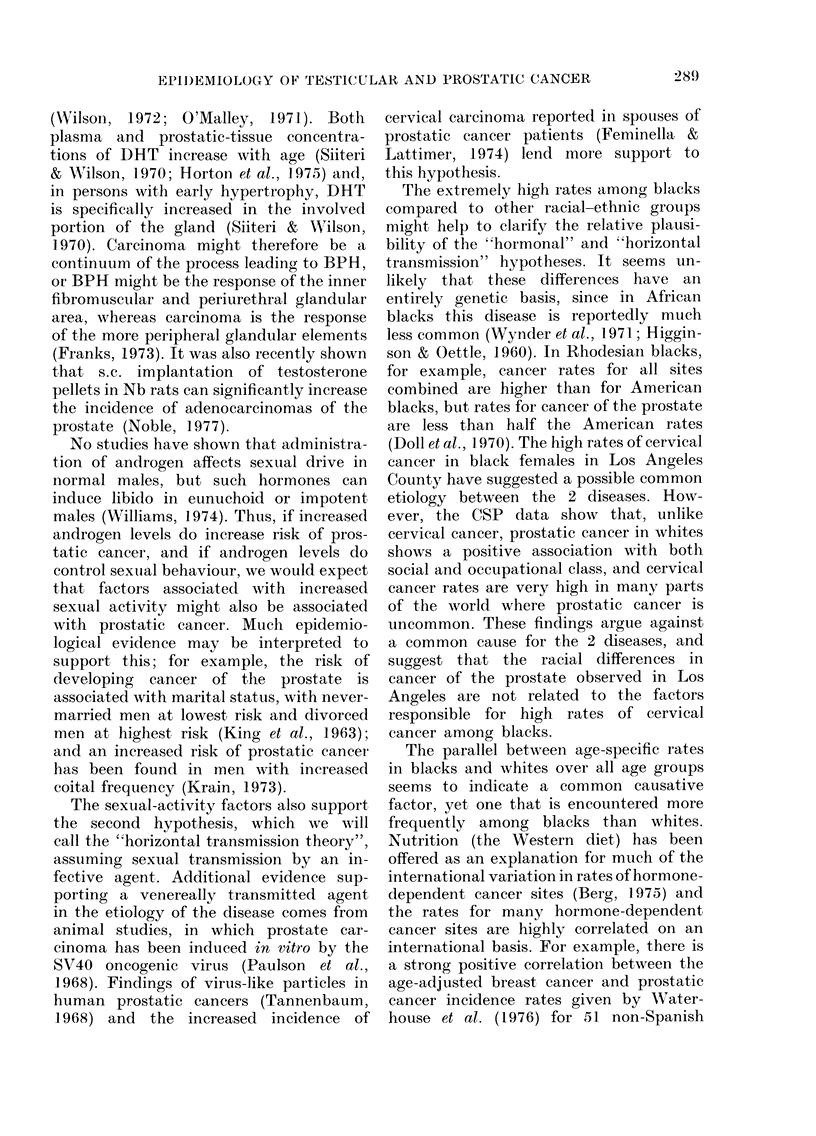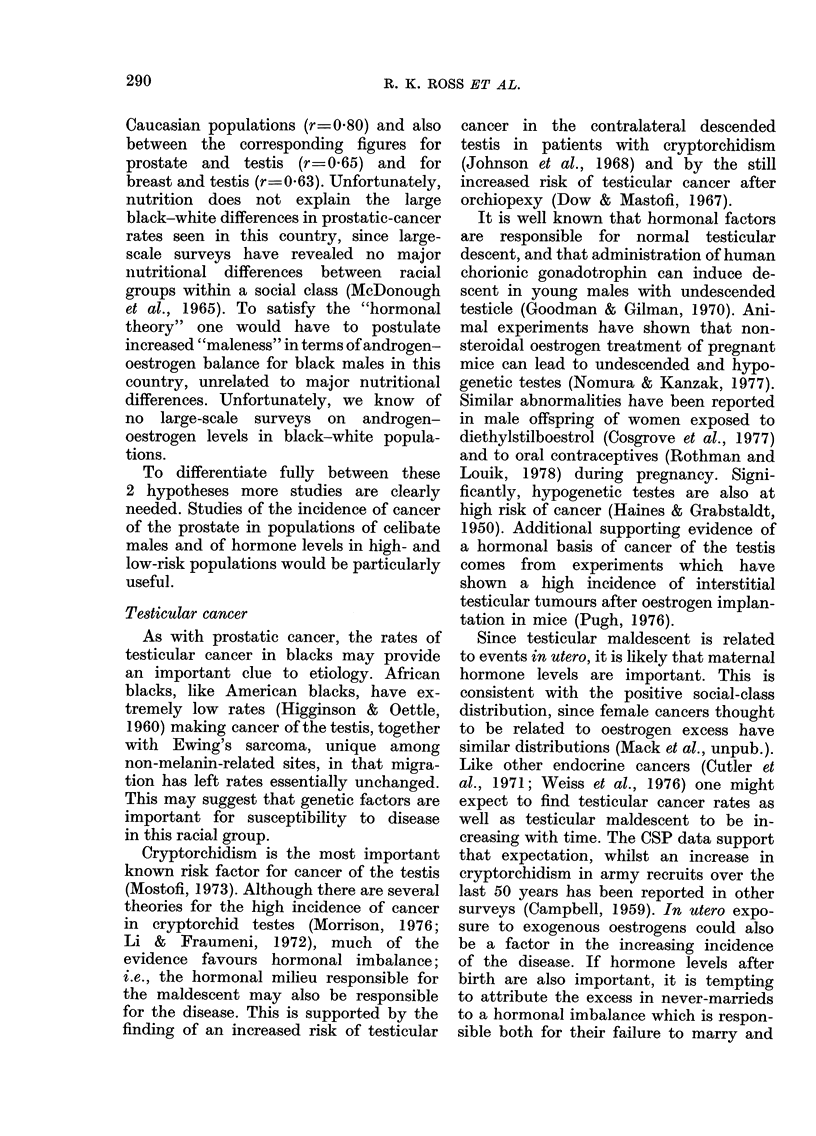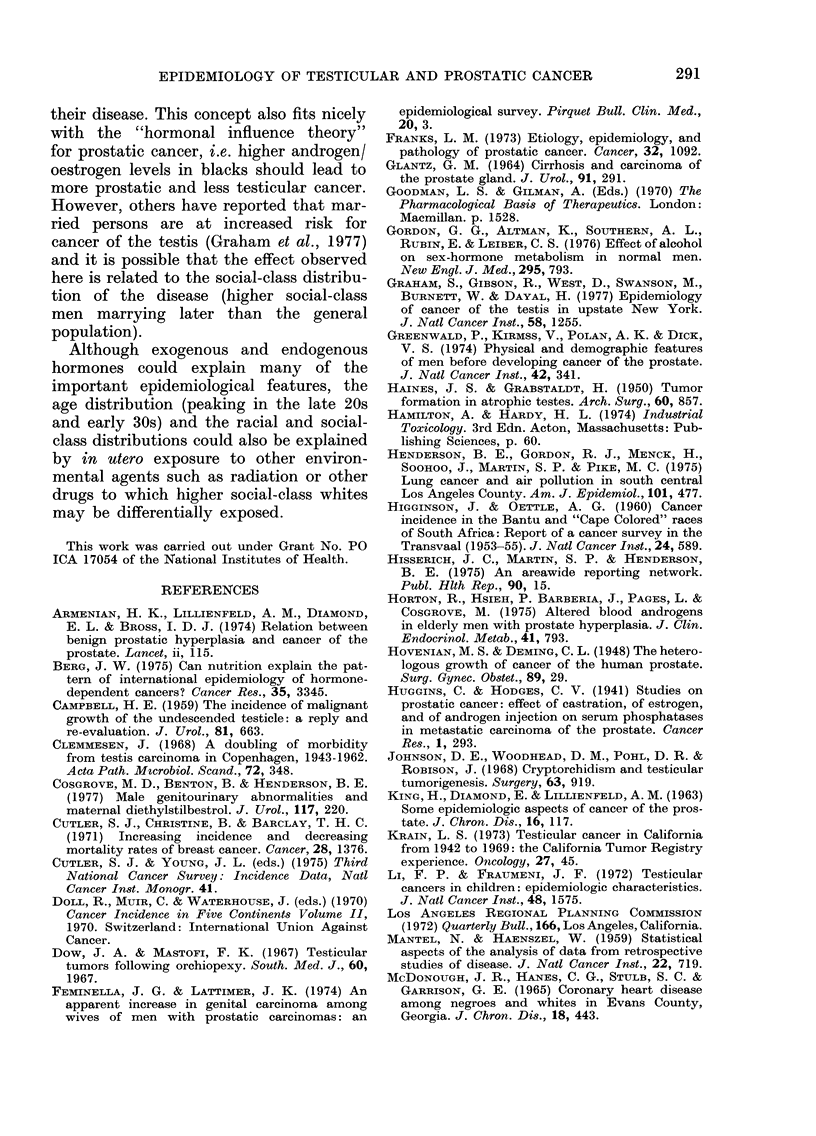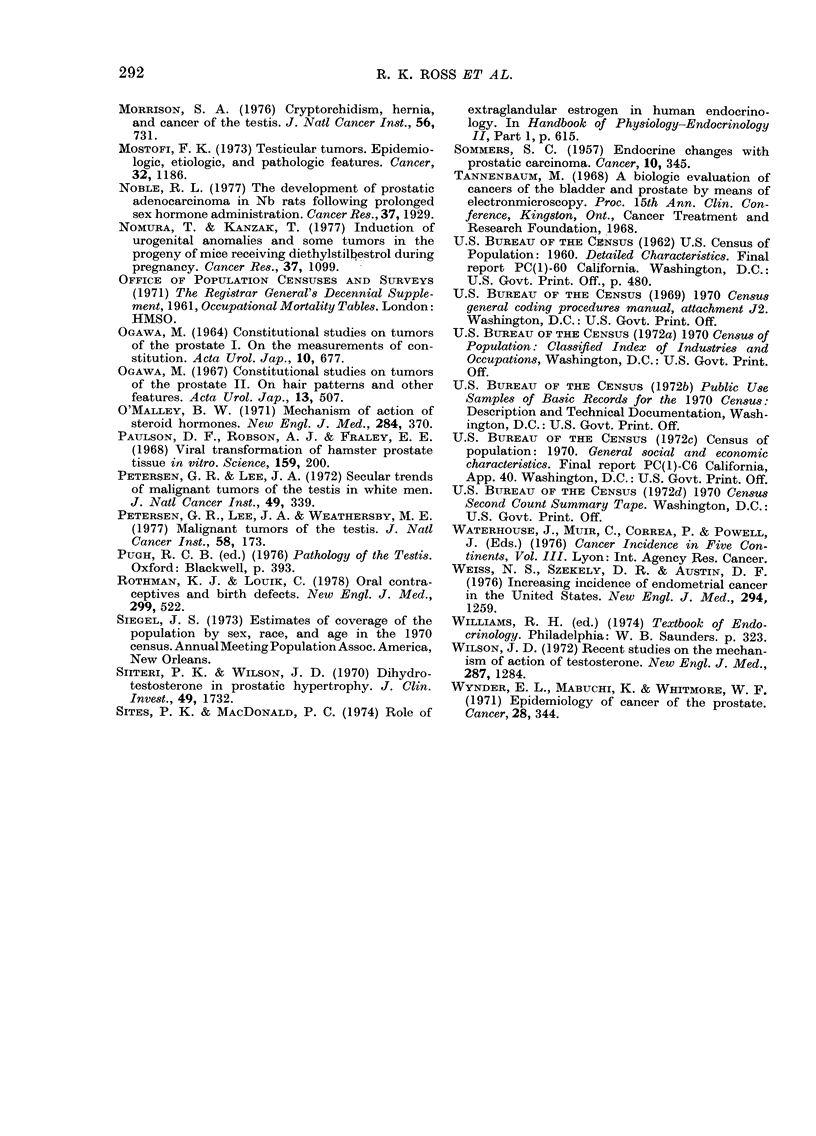# Descriptive epidemiology of testicular and prostatic cancer in Los Angeles.

**DOI:** 10.1038/bjc.1979.53

**Published:** 1979-03

**Authors:** R. K. Ross, J. W. McCurtis, B. E. Henderson, H. R. Menck, T. M. Mack, S. P. Martin

## Abstract

Data from the Los Angeles County Cancer Surveillance Program (CSP) from 1972 to 1975 were used to study the descriptive epidemiology of testicular cancer and prostatic cancer. The very high black/white ratio and late age peak of cancer of the prostate contrasted sharply with the very low ratio and early age peak of testicular cancer. However, both sites had higher rates among upper occupational and social class groupings. Avalable descriptive and analytical research suggests that the etiology of prostatic cancer is most probably related to hormonal influences rather than to a horizontally transmitted agent, while the etiology of testicular cancer is most probably related to endogenous or exogenous hormonal influences in utero or in infancy, or to in utero exposure to other exogenous agents.


*Br. J. Cancer* (1979) **39**, 284

### DESCRIPTIVE EPIDEMIOLOGY OF TESTICULAR AND PROSTATIC CANCER IN LOS ANGELES

R. K. ROSS,* J. W. McCURTIS,† B. E. HENDERSON,* H. R. MENCK,‡ T. M. MACK,‡ AND S. P. MARTIN‡

*From the *Department of Community and Family Medicine, University of Southern California School of Medicine,* 2025 *Zonal Ave., Los Angeles, California* 90033, *the †Department of Sociology, California State University, Dominguez Hills, California and the ‡Cancer Surveillance Program, University of Southern California School of Medicine, Los Angeles, California*

Received 18 August 1978 Accepted 17 November 1978

**Summary.—Data from the Los Angeles County Cancer Surveillance Program (CSP) from 1972 to 1975 were used to study the descriptive epidemiology of testicular cancer and prostatic cancer. The very high black/white ratio and late age peak of cancer of the prostate contrasted sharply with the very low ratio and early age peak of testicular cancer. However, both sites had higher rates among upper occupational and social class groupings. Available descriptive and analytical research suggests that the etiology of prostatic cancer is most probably related to hormonal influences rather than to a horizontally transmitted agent, while the etiology of testicular cancer is most probably related to endogenous or exogenous hormonal influences *in utero* or in infancy, or to *in utero* exposure to other exogenous agents.**

There is a large body of medical literature on the epidemiology of female genital cancer, and many risk factors for these diseases are carefully defined. Studies of male genital cancer, particularly of the testis and prostate, have been less frequent, despite the fact that testicular cancer rates have substantially increased in recent years (Clemmesen, 1968; Petersen & Lee, 1972) and that in the United States the prostate is already the second commonest site of cancer in the male (Cutler & Young, 1975). Prostatic cancer rates have also been reported to be rising in various populations (Wynder *et al.*, 1971; Krain, 1973). In fact, age-adjusted prostatic cancer rates for black males in Los Angeles County are higher than the rates for any other site for either sex or any racial–ethnic group.

Descriptive epidemiological data from a population-based Cancer Surveillance Program can be readily used as an initial approach in forming and testing hypotheses. With the aid of census data to provide denominators when available, we have begun to explore such variables as occupation, marital status, social class, histological type, age, sex, race, and secular trend on a site-by-site basis in Los Angeles County. We report here our current findings of 2 sites, prostate and testis, remarkable for their contrasts, yet possessing several common characteristics. Since the black and white populations are larger than the other racial–ethnic groups, much of the discussion will be limited to these 2 groups.

### MATERIALS AND METHODS

The Cancer Surveillance Program (CSP), a comprehensive population-based cancer registry of Los Angeles County, has now accumulated 4 years' incidence data (1972–1975). The methodology has been discussed elsewhere (Hisserich *et al.*, 1975). The CSP demographic data, including occupation and industry, age, race, sex, and marital status, are abstracted by trained medical record technicians from hospital admission sheets and from medical records. As in the 1970 census, whites are divided into "Mexican-Americans" and "other white" (non-Spanish)

categories using the detailed Spanish surname list, including optional name endings (U.S. Bureau of the Census, 1969). Occupation and industry are coded into one of 417 occupational codes and one of 215 industry codes, using the 1970 U.S. Census classification system (U.S. Bureau of the Census, 1972*a*). Populations-at-risk for computing expected number of cases by occupation and industry are calculated from Public Use Samples of Basic Records from the 1970 Census for Los Angeles County: a 1-in-50 sampling of the 7 million Los Angeles County residents (U.S. Bureau of the Census, 1972*b*). Denominator data for calculating expected numbers by marital status are likewise obtained from 1970 Census data for Los Angeles County (U.S. Bureau of the Census, 1972*c*).

Unlike other variables collected by the CSP, social class (SC) is assigned on a geographical not an individual basis, as determined by census tract of residence at the time of diagnosis, using the two-factor Hollingshead Index (Henderson *et al.*, 1975). Since other cancer sites with well-known social-class patterns (*e.g.* breast and cervix) show the expected social-class distributions, we feel it is unlikely that this method introduces systematic errors in studying broad social-class categories. However, efforts to study the social-class distribution in blacks by this method have proved more difficult, apparently owing to upward social-class mobility by segments of the black population since the 1970 Census; *i.e.*, black populations in the upper social-class census tracts are substantially underestimated.

The CSP makes adjustments of census data to allow for intercensal growth and undercounting (Siegel, 1973). Zero growth is assumed for whites (Los Angeles Regional Planning Commission, 1972) whilst the average annual age-specific rates of population change for Mexican-Americans and blacks, 1960 (U.S. Bureau of the Census, 1962) to 1970 (U.S. Bureau of the Census, 1972*d*) are applied for the interval between the 1970 Census and January 1, 1974, to adjust for postcensal growth within these groups.

Age-adjusted incidence rates are calculated by the direct method using 10-year age groupings from the U.S. 1970 population as the standard. A summary $\chi^2$ test (Mantel & Haenszel, 1959) is used to measure statistical significance.

### RESULTS

Perhaps the most striking finding in examining the 2 diseases simultaneously is the virtual absence of testicular cancer among blacks in Los Angeles County, while prostatic cancer among blacks is twice as frequent as among the other racial–ethnic groups (Table I). The large rate

FIG. 1—Average annual prostatic (P) and testicular (T) cancer age-specific incidence rates by race, Los Angeles County, 1972–1975.

TABLE I.—*Prostatic and testicular cancer: average annual age-adjusted incidence rates per* 100,000 *by race, Los Angeles County,* 1972–1975 (*No. of cases in parentheses*)

| | Testis | $\chi^2$ | Prostate | $\chi^2$ |
|---|---|---|---|---|
| Black | 0·69 (11) | 36·3** | 117·45 (792) | 354·7** |
| Other white (Non-Spanish surnamed) | 3·37 (360) | — | 59·29 (5357) | — |
| Mexican-American | 2·16 (56) | 8·1** | 58·62 (477) | 0·6 |

** $P<0·01$ compared to other whites differences between racial groups hold at all age groups for both sites. The characteristic rapid increase in prostatic cancer rates begins at age 40, in sharp contrast to the 25–35 peak age for testicular cancer (Fig. 1, testicular cancer in blacks omitted since there were only 11 cases).

We continue to see increasing rates in Los Angeles County in the 1970s for cancer of the testis in whites (Fig. 2). Mexican-Americans and blacks show large variations between periods, owing to small numbers, but there is yet no evidence that the testicular-cancer rates in these groups are also increasing. Prostatic cancer rates are more stable, and trends are therefore clearer. Black prostatic cancer rates have shown a modest but steady increase. Mexican-American rates, on the other hand, have increased more substantially and appear to have caught up with and surpassed those of whites, whose rates have shown a slight but steady decline (Fig. 2).

Although the results of previous studies of cancer of the prostate by social class (SC) have been inconsistent (King *et al.*, 1963; Office of Population Census Surveys, 1971) we find a higher risk among higher social classes with overall age-adjusted rates in whites in SCI (high social class), 23% higher than in SCV (low social class) (Table IIa). Table IIa also shows the social-class distribution for cancer of the testis in whites, and an even greater gradient is apparent. Although numbers

TABLE IIa.—*Average annual age-adjusted incidence rates of prostatic and testicular cancer by social class, Los Angeles County,* 1972–1975 (*No. of cases in parentheses*)

| | Whites | |
|---|---|---|
| Social class | Prostate | Testis |
| I (highest) | 72·80 (397) | 5·10 (49) |
| II | 64·10 (1221) | 3·34 (88) |
| III | 58·68 (1323) | 3·83 (105) |
| IV | 54·92 (1884) | 3·15 (103) |
| V (lowest) | 59·10 (496) | 2·02 (12) |
| Total | 59·29 | 3·37 |

FIG. 2—Prostatic (P) and testicular (T) cancer age-adjusted incidence rates by race and year, Los Angeles County, 1972–1975 (number of cases annually in parentheses).

FIG. 3—Testicular cancer age-specific incidence rates by social class: whites only, Los Angeles County, 1972–1975.

are very small, all social-class groups for testicular cancer except I and V are bimodal by age, with peaks in the 25–35 age interval and again after the age of 75. Social Class I does not have a second peak, and Social Class V has a single peak in the interval 45–55 (Fig. 3). The significance of this delayed peak in the lowest social class is unclear, but has been previously reported (Petersen *et al.*, 1977).

Given the problem of estimating denominators in the higher social classes of the black population and in the absence of data on incidence before 1972, we looked at prostatic cancer mortality using the same social-class criteria for blacks and whites in Los Angeles County from 1969 to 1971 (Table IIb). Neither a positive nor negative social-class trend is apparent for either blacks or whites. It seems unlikely from these results that much of the racial differences in prostatic cancer rates is explained by social-class differences between the races. Failure to find a positive social-class trend in whites (and blacks) could be related to different survival rates among the different classes.

TABLE IIb.—*Age-adjusted prostatic cancer mortality rates by social class and race, Los Angeles County,* 1969–1971* (*No. of deaths in parentheses*)

| Social class | Whites | Blacks |
|---|---|---|
| I (highest) | 19·3 (69) | — (0) |
| II | 17·7 (242) | 35·2 (5) |
| III | 18·8 (310) | 37·7 (11) |
| IV | 18·8 (482) | 29·5 (61) |
| V (lowest) | 15·7 (105) | 33·2 (72) |
| Unknown | (30) | (7) |

* From Los Angeles County death certificates provided by Mr Martin Donabedian of the Los Angeles County Department of Health.

When grouped into large occupational subcategories, testicular and prostatic cancer appear remarkably similar (Table III). These groupings offer further evidence of a positive social-class distribution for the 2 diseases, since "white collar" subgroupings such as professionals and managers have more cancer than expected, while "blue collar" occupations such as labourers and operatives have less than expected. Cadmium exposure has been suggested as an etiological agent for prostatic cancer. Occupations involving possible cadmium exposure such as welding, painting, metal plating and photography (Hamilton & Hardy, 1974) do not show significantly higher than expected prostatic cancer rates in Los Angeles County during the study period (Table IV).

TABLE III.—*Distribution of testicular and prostatic cancer by occupational subgroup for whites, Los Angeles County,* 1972–1975 (*under* 65 *only*)

| Occupational subgroup* | Testis Obs/expt† (No. of cases) | Prostate Obs/expt† (No. of cases) |
|---|---|---|
| Professionals | 1·41 (71)** | 1·51 (185)** |
| Managers | 1·52 (44)** | 1·57 (203)** |
| Salesmen | 1·15 (22) | 1·22 (96) |
| Clerical | 0·76 (17) | 0·79 (49) |
| Craftsmen | 0·90 (51) | 0·73 (156)** |
| Operatives | 0·31 (9)** | 0·60 (53)** |
| Transportation workers | 0·99 (12) | 0·84 (30) |
| Labourers | 0·66 (8) | 0·94 (35) |
| Servicers | 0·84 (14) | 0·89 (63) |

* Includes all occupations except retired and unknown.
† Expected based on age-specific rates for all occupations applied to populations-at-risk.
** $P<0·01$.

TABLE IV.—*Distribution of prostatic cancer in whites by occupations involving possible cadmium exposure, Los Angeles County,* 1972–1975 (*under* 65 *only*)

| Occupation | No. | SIR† |
|---|---|---|
| Photographer | 4 | 171·8 |
| Painter | 11 | 68·3 |
| Welder/Solderer | 9 | 95·9 |
| Metal Plating | 1 | 124·1 |
| Total | 25 | 87·3 |

† Standard Incidence Ratio = (Observed number of cases ÷ expected number of cases, based on overall occupation distribution in white males in Los Angeles County) × 100; $P<0·05$ for all.

The ratio of age-adjusted prostatic cancer rates in ever-married (EM, 4898 cases) to never-married (NM, 332 cases) white males in Los Angeles County is 1·2, in contrast to that for cancer of the

TABLE V.—*A comparison of prostatic and testicular cancer in Los Angeles County, 1972–1975*

| | Prostate | Testis |
|---|---|---|
| Black/white age-adjusted ratio | 2·0 | 0·2 |
| Age | Peaks after 75 | Peaks before 35 |
| Secular trend (whites only) | Decreasing | Increasing |
| Ever married/never married | 1·2 | 0·7 |
| Social class (whites only) | + Association | + + Association |
| Occupation (whites only) | White collar | White collar |

testis, which is more common among never-marrieds (EM/NM=0·7; 231 married, 123 never married). By age, these effects are limited to the highest-risk age groups (for prostate, the ratio in men less than 65 is 1·00, while for testis the ratio is 1·51 in men under the age of 25 and 0·59 for the 25–55 age groups combined).

Although overall testicular cancer rates peak in the 25–35 age group, this is not consistently so for all histological types. As expected, embryonal carcinomas (38% of all cases) and teratocarcinomas (9% of all cases) peak considerably earlier than seminomas (48% of cases) in this series (under 25 in the former, compared to a broad peak in the 25–45 age groups for the latter). The deficit of cancer of the testis in blacks is present for all 3 major histological types; however, in Mexican–Americans the age-adjusted rates for embryonal carcinomas are actually similar to rates in whites. Although there is a suggestion of a positive social-class trend for both seminomas and embryonal carcinomas, the effect is stronger for the latter histological type (ratio of highest SC to lowest is 1·7 for seminomas but 5·7 for embryonal carcinomas). Teratocarcinomas could not be analysed by social class because of the small number of cases.

Table V summarizes the similarities and contrasts in the descriptive epidemiology of prostatic and testicular cancer in Los Angeles County.

### DISCUSSION

### *Prostatic cancer*

We feel that 2 major hypotheses concerning the etiology of cancer of the prostate have been most strongly corroborated by research findings. The first we will call the "hormonal influence theory", assuming an abnormal hormonal environment as the requisite for development of disease. Several observations suggest that androgens and oestrogens may be involved in the pathogenesis of the disease. Firstly, necropsy studies show that patients with cirrhosis of the liver have less prostatic cancer than controls (Glantz, 1964). Alcohol depresses testosterone levels (Gordon *et al.*, 1976) and liver disease leads to hyperoestrogenism (Sites & MacDonald, 1974). The studies of Ogawa (1964, 1967) and Wynder *et al.* (1971) also lend support to this hypothesis. They found that patients with prostatic cancer tend to have more body hair and to be less obese than controls. Greenwald, however, studying anthropometric indices in 268 college men who eventually developed prostatic cancer, found no difference in such variables as somatotype, baldness, and gynandromorphy compared to controls (Greenwald *et al.*, 1974). Also both castration and oestrogen therapy have a palliative effect on advanced prostatic cancer (Huggins & Hodges, 1941) and prostatic cancer is seemingly unknown in castrates (Hovenian & Deming, 1948). Furthermore several studies have shown an association between benign prostatic hypertrophy (BPH) and prostatic cancer (Armenian *et al.*, 1974; Sommers, 1957), which suggests a role for dihydrotestosterone (DHT) in the pathogenesis of the disease. DHT is the major androgen promoting growth of prostatic tissue, having been derived from testosterone in the endoplasmic reticulum or nuclear membrane of prostate cells

(Wilson, 1972; O'Malley, 1971). Both plasma and prostatic-tissue concentrations of DHT increase with age (Siiteri & Wilson, 1970; Horton *et al.*, 1975) and, in persons with early hypertrophy, DHT is specifically increased in the involved portion of the gland (Siiteri & Wilson, 1970). Carcinoma might therefore be a continuum of the process leading to BPH, or BPH might be the response of the inner fibromuscular and periurethral glandular area, whereas carcinoma is the response of the more peripheral glandular elements (Franks, 1973). It was also recently shown that s.c. implantation of testosterone pellets in Nb rats can significantly increase the incidence of adenocarcinomas of the prostate (Noble, 1977).

No studies have shown that administration of androgen affects sexual drive in normal males, but such hormones can induce libido in eunuchoid or impotent males (Williams, 1974). Thus, if increased androgen levels do increase risk of prostatic cancer, and if androgen levels do control sexual behaviour, we would expect that factors associated with increased sexual activity might also be associated with prostatic cancer. Much epidemiological evidence may be interpreted to support this; for example, the risk of developing cancer of the prostate is associated with marital status, with never-married men at lowest risk and divorced men at highest risk (King *et al.*, 1963); and an increased risk of prostatic cancer has been found in men with increased coital frequency (Krain, 1973).

The sexual-activity factors also support the second hypothesis, which we will call the "horizontal transmission theory", assuming sexual transmission by an infective agent. Additional evidence supporting a venereally transmitted agent in the etiology of the disease comes from animal studies, in which prostate carcinoma has been induced *in vitro* by the SV40 oncogenic virus (Paulson *et al.*, 1968). Findings of virus-like particles in human prostatic cancers (Tannenbaum, 1968) and the increased incidence of cervical carcinoma reported in spouses of prostatic cancer patients (Feminella & Lattimer, 1974) lend more support to this hypothesis.

The extremely high rates among blacks compared to other racial–ethnic groups might help to clarify the relative plausibility of the "hormonal" and "horizontal transmission" hypotheses. It seems unlikely that these differences have an entirely genetic basis, since in African blacks this disease is reportedly much less common (Wynder *et al.*, 1971; Higginson & Oettle, 1960). In Rhodesian blacks, for example, cancer rates for all sites combined are higher than for American blacks, but rates for cancer of the prostate are less than half the American rates (Doll *et al.*, 1970). The high rates of cervical cancer in black females in Los Angeles County have suggested a possible common etiology between the 2 diseases. However, the CSP data show that, unlike cervical cancer, prostatic cancer in whites shows a positive association with both social and occupational class, and cervical cancer rates are very high in many parts of the world where prostatic cancer is uncommon. These findings argue against a common cause for the 2 diseases, and suggest that the racial differences in cancer of the prostate observed in Los Angeles are not related to the factors responsible for high rates of cervical cancer among blacks.

The parallel between age-specific rates in blacks and whites over all age groups seems to indicate a common causative factor, yet one that is encountered more frequently among blacks than whites. Nutrition (the Western diet) has been offered as an explanation for much of the international variation in rates of hormone-dependent cancer sites (Berg, 1975) and the rates for many hormone-dependent cancer sites are highly correlated on an international basis. For example, there is a strong positive correlation between the age-adjusted breast cancer and prostatic cancer incidence rates given by Waterhouse *et al.* (1976) for 51 non-Spanish

Caucasian populations ($r=0{\cdot}80$) and also between the corresponding figures for prostate and testis ($r=0{\cdot}65$) and for breast and testis ($r=0{\cdot}63$). Unfortunately, nutrition does not explain the large black–white differences in prostatic-cancer rates seen in this country, since large-scale surveys have revealed no major nutritional differences between racial groups within a social class (McDonough *et al.*, 1965). To satisfy the "hormonal theory" one would have to postulate increased "maleness" in terms of androgen–oestrogen balance for black males in this country, unrelated to major nutritional differences. Unfortunately, we know of no large-scale surveys on androgen–oestrogen levels in black–white populations.

To differentiate fully between these 2 hypotheses more studies are clearly needed. Studies of the incidence of cancer of the prostate in populations of celibate males and of hormone levels in high- and low-risk populations would be particularly useful.

*Testicular cancer*

As with prostatic cancer, the rates of testicular cancer in blacks may provide an important clue to etiology. African blacks, like American blacks, have extremely low rates (Higginson & Oettle, 1960) making cancer of the testis, together with Ewing's sarcoma, unique among non-melanin-related sites, in that migration has left rates essentially unchanged. This may suggest that genetic factors are important for susceptibility to disease in this racial group.

Cryptorchidism is the most important known risk factor for cancer of the testis (Mostofi, 1973). Although there are several theories for the high incidence of cancer in cryptorchid testes (Morrison, 1976; Li & Fraumeni, 1972), much of the evidence favours hormonal imbalance; *i.e.*, the hormonal milieu responsible for the maldescent may also be responsible for the disease. This is supported by the finding of an increased risk of testicular cancer in the contralateral descended testis in patients with cryptorchidism (Johnson *et al.*, 1968) and by the still increased risk of testicular cancer after orchiopexy (Dow & Mastofi, 1967).

It is well known that hormonal factors are responsible for normal testicular descent, and that administration of human chorionic gonadotrophin can induce descent in young males with undescended testicle (Goodman & Gilman, 1970). Animal experiments have shown that non-steroidal oestrogen treatment of pregnant mice can lead to undescended and hypogenetic testes (Nomura & Kanzak, 1977). Similar abnormalities have been reported in male offspring of women exposed to diethylstilboestrol (Cosgrove *et al.*, 1977) and to oral contraceptives (Rothman and Louik, 1978) during pregnancy. Significantly, hypogenetic testes are also at high risk of cancer (Haines & Grabstaldt, 1950). Additional supporting evidence of a hormonal basis of cancer of the testis comes from experiments which have shown a high incidence of interstitial testicular tumours after oestrogen implantation in mice (Pugh, 1976).

Since testicular maldescent is related to events *in utero*, it is likely that maternal hormone levels are important. This is consistent with the positive social-class distribution, since female cancers thought to be related to oestrogen excess have similar distributions (Mack *et al.*, unpub.). Like other endocrine cancers (Cutler *et al.*, 1971; Weiss *et al.*, 1976) one might expect to find testicular cancer rates as well as testicular maldescent to be increasing with time. The CSP data support that expectation, whilst an increase in cryptorchidism in army recruits over the last 50 years has been reported in other surveys (Campbell, 1959). *In utero* exposure to exogenous oestrogens could also be a factor in the increasing incidence of the disease. If hormone levels after birth are also important, it is tempting to attribute the excess in never-marrieds to a hormonal imbalance which is responsible both for their failure to marry and their disease. This concept also fits nicely with the "hormonal influence theory" for prostatic cancer, *i.e.* higher androgen/oestrogen levels in blacks should lead to more prostatic and less testicular cancer. However, others have reported that married persons are at increased risk for cancer of the testis (Graham *et al.*, 1977) and it is possible that the effect observed here is related to the social-class distribution of the disease (higher social-class men marrying later than the general population).

Although exogenous and endogenous hormones could explain many of the important epidemiological features, the age distribution (peaking in the late 20s and early 30s) and the racial and social-class distributions could also be explained by *in utero* exposure to other environmental agents such as radiation or other drugs to which higher social-class whites may be differentially exposed.

This work was carried out under Grant No. PO ICA 17054 of the National Institutes of Health.

### REFERENCES

ARMENIAN, H. K., LILLIENFELD, A. M., DIAMOND, E. L. & BROSS, I. D. J. (1974) Relation between benign prostatic hyperplasia and cancer of the prostate. *Lancet*, ii, 115.

BERG, J. W. (1975) Can nutrition explain the pattern of international epidemiology of hormone-dependent cancers? *Cancer Res.*, **35**, 3345.

CAMPBELL, H. E. (1959) The incidence of malignant growth of the undescended testicle: a reply and re-evaluation. *J. Urol.*, **81**, 663.

CLEMMESEN, J. (1968) A doubling of morbidity from testis carcinoma in Copenhagen, 1943-1962. *Acta Path. Microbiol. Scand.*, **72**, 348.

COSGROVE, M. D., BENTON, B. & HENDERSON, B. E. (1977) Male genitourinary abnormalities and maternal diethylstilbestrol. *J. Urol.*, **117**, 220.

CUTLER, S. J., CHRISTINE, B. & BARCLAY, T. H. C. (1971) Increasing incidence and decreasing mortality rates of breast cancer. *Cancer*, **28**, 1376.

CUTLER, S. J. & YOUNG, J. L. (eds.) (1975) *Third National Cancer Survey: Incidence Data, Natl Cancer Inst. Monogr.* **41**.

DOLL, R., MUIR, C. & WATERHOUSE, J. (eds.) (1970) *Cancer Incidence in Five Continents Volume II*, 1970. Switzerland: International Union Against Cancer.

DOW, J. A. & MASTOFI, F. K. (1967) Testicular tumors following orchiopexy. *South. Med. J.*, **60**, 1967.

FEMINELLA, J. G. & LATTIMER, J. K. (1974) An apparent increase in genital carcinoma among wives of men with prostatic carcinomas: an epidemiological survey. *Pirquet Bull. Clin. Med.*, **20**, 3.

FRANKS, L. M. (1973) Etiology, epidemiology, and pathology of prostatic cancer. *Cancer*, **32**, 1092.

GLANTZ, G. M. (1964) Cirrhosis and carcinoma of the prostate gland. *J. Urol.*, **91**, 291.

GOODMAN, L. S. & GILMAN, A. (Eds.) (1970) *The Pharmacological Basis of Therapeutics*. London: Macmillan. p. 1528.

GORDON, G. G., ALTMAN, K., SOUTHERN, A. L., RUBIN, E. & LEIBER, C. S. (1976) Effect of alcohol on sex-hormone metabolism in normal men. *New Engl. J. Med.*, **295**, 793.

GRAHAM, S., GIBSON, R., WEST, D., SWANSON, M., BURNETT, W. & DAYAL, H. (1977) Epidemiology of cancer of the testis in upstate New York. *J. Natl Cancer Inst.*, **58**, 1255.

GREENWALD, P., KIRMSS, V., POLAN, A. K. & DICK, V. S. (1974) Physical and demographic features of men before developing cancer of the prostate. *J. Natl Cancer Inst.*, **42**, 341.

HAINES, J. S. & GRABSTALDT, H. (1950) Tumor formation in atrophic testes. *Arch. Surg.*, **60**, 857.

HAMILTON, A. & HARDY, H. L. (1974) *Industrial Toxicology*. 3rd Edn. Acton, Massachusetts: Publishing Sciences, p. 60.

HENDERSON, B. E., GORDON, R. J., MENCK, H., SOOHOO, J., MARTIN, S. P. & PIKE, M. C. (1975) Lung cancer and air pollution in south central Los Angeles County. *Am. J. Epidemiol.*, **101**, 477.

HIGGINSON, J. & OETTLE, A. G. (1960) Cancer incidence in the Bantu and "Cape Colored" races of South Africa: Report of a cancer survey in the Transvaal (1953–55). *J. Natl Cancer Inst.*, **24**, 589.

HISSERICH, J. C., MARTIN, S. P. & HENDERSON, B. E. (1975) An areawide reporting network. *Publ. Hlth Rep.*, **90**, 15.

HORTON, R., HSIEH, P. BARBERIA, J., PAGES, L. & COSGROVE, M. (1975) Altered blood androgens in elderly men with prostate hyperplasia. *J. Clin. Endocrinol. Metab.*, **41**, 793.

HOVENIAN, M. S. & DEMING, C. L. (1948) The heterologous growth of cancer of the human prostate. *Surg. Gynec. Obstet.*, **89**, 29.

HUGGINS, C. & HODGES, C. V. (1941) Studies on prostatic cancer: effect of castration, of estrogen, and of androgen injection on serum phosphatases in metastatic carcinoma of the prostate. *Cancer Res.*, **1**, 293.

JOHNSON, D. E., WOODHEAD, D. M., POHL, D. R. & ROBISON, J. (1968) Cryptorchidism and testicular tumorigenesis. *Surgery*, **63**, 919.

KING, H., DIAMOND, E. & LILLIENFELD, A. M. (1963) Some epidemiologic aspects of cancer of the prostate. *J. Chron. Dis.*, **16**, 117.

KRAIN, L. S. (1973) Testicular cancer in California from 1942 to 1969: the California Tumor Registry experience. *Oncology*, **27**, 45.

LI, F. P. & FRAUMENI, J. F. (1972) Testicular cancers in children: epidemiologic characteristics. *J. Natl Cancer Inst.*, **48**, 1575.

LOS ANGELES REGIONAL PLANNING COMMISSION (1972) *Quarterly Bull.*, **166**, Los Angeles, California.

MANTEL, N. & HAENSZEL, W. (1959) Statistical aspects of the analysis of data from retrospective studies of disease. *J. Natl Cancer Inst.*, **22**, 719.

MCDONOUGH, J. R., HANES, C. G., STULB, S. C. & GARRISON, G. E. (1965) Coronary heart disease among negroes and whites in Evans County, Georgia. *J. Chron. Dis.*, **18**, 443.

MORRISON, S. A. (1976) Cryptorchidism, hernia, and cancer of the testis. *J. Natl Cancer Inst.*, **56**, 731.

MOSTOFI, F. K. (1973) Testicular tumors. Epidemiologic, etiologic, and pathologic features. *Cancer*, **32**, 1186.

NOBLE, R. L. (1977) The development of prostatic adenocarcinoma in Nb rats following prolonged sex hormone administration. *Cancer Res.*, **37**, 1929.

NOMURA, T. & KANZAK, T. (1977) Induction of urogenital anomalies and some tumors in the progeny of mice receiving diethylstilbestrol during pregnancy. *Cancer Res.*, **37**, 1099.

OFFICE OF POPULATION CENSUSES AND SURVEYS (1971) *The Registrar General's Decennial Supplement*, 1961, *Occupational Mortality Tables*. London: HMSO.

OGAWA, M. (1964) Constitutional studies on tumors of the prostate I. On the measurements of constitution. *Acta Urol. Jap.*, **10**, 677.

OGAWA, M. (1967) Constitutional studies on tumors of the prostate II. On hair patterns and other features. *Acta Urol. Jap.*, **13**, 507.

O'MALLEY, B. W. (1971) Mechanism of action of steroid hormones. *New Engl. J. Med.*, **284**, 370.

PAULSON, D. F., ROBSON, A. J. & FRALEY, E. E. (1968) Viral transformation of hamster prostate tissue *in vitro*. *Science*, **159**, 200.

PETERSEN, G. R. & LEE, J. A. (1972) Secular trends of malignant tumors of the testis in white men. *J. Natl Cancer Inst.*, **49**, 339.

PETERSEN, G. R., LEE, J. A. & WEATHERSBY, M. E. (1977) Malignant tumors of the testis. *J. Natl Cancer Inst.*, **58**, 173.

PUGH, R. C. B. (ed.) (1976) *Pathology of the Testis*. Oxford: Blackwell, p. 393.

ROTHMAN, K. J. & LOUIK, C. (1978) Oral contraceptives and birth defects. *New Engl. J. Med.*, **299**, 522.

SIEGEL, J. S. (1973) Estimates of coverage of the population by sex, race, and age in the 1970 census. Annual Meeting Population Assoc. America, New Orleans.

SIITERI, P. K. & WILSON, J. D. (1970) Dihydrotestosterone in prostatic hypertrophy. *J. Clin. Invest.*, **49**, 1732.

SITES, P. K. & MACDONALD, P. C. (1974) Role of extraglandular estrogen in human endocrinology. In *Handbook of Physiology–Endocrinology II*, Part 1, p. 615.

SOMMERS, S. C. (1957) Endocrine changes with prostatic carcinoma. *Cancer*, **10**, 345.

TANNENBAUM, M. (1968) A biologic evaluation of cancers of the bladder and prostate by means of electronmicroscopy. *Proc. 15th Ann. Clin. Conference, Kingston, Ont.*, Cancer Treatment and Research Foundation, 1968.

U.S. BUREAU OF THE CENSUS (1962) U.S. Census of Population: 1960. *Detailed Characteristics*. Final report PC(1)-60 California. Washington, D.C.: U.S. Govt. Print. Off., p. 480.

U.S. BUREAU OF THE CENSUS (1969) 1970 *Census general coding procedures manual, attachment J2*. Washington, D.C.: U.S. Govt. Print. Off.

U.S. BUREAU OF THE CENSUS (1972*a*) 1970 *Census of Population: Classified Index of Industries and Occupations*, Washington, D.C.: U.S. Govt. Print. Off.

U.S. BUREAU OF THE CENSUS (1972*b*) *Public Use Samples of Basic Records for the* 1970 *Census:* Description and Technical Documentation, Washington, D.C.: U.S. Govt. Print. Off.

U.S. BUREAU OF THE CENSUS (1972*c*) Census of population: 1970. *General social and economic characteristics*. Final report PC(1)-C6 California, App. 40. Washington, D.C.: U.S. Govt. Print. Off.

U.S. BUREAU OF THE CENSUS (1972*d*) 1970 *Census Second Count Summary Tape*. Washington, D.C.: U.S. Govt. Print. Off.

WATERHOUSE, J., MUIR, C., CORREA, P. & POWELL, J. (Eds.) (1976) *Cancer Incidence in Five Continents, Vol. III*. Lyon: Int. Agency Res. Cancer.

WEISS, N. S., SZEKELY, D. R. & AUSTIN, D. F. (1976) Increasing incidence of endometrial cancer in the United States. *New Engl. J. Med.*, **294**, 1259.

WILLIAMS, R. H. (ed.) (1974) *Textbook of Endocrinology*. Philadelphia: W. B. Saunders. p. 323.

WILSON, J. D. (1972) Recent studies on the mechanism of action of testosterone. *New Engl. J. Med.*, **287**, 1284.

WYNDER, E. L., MABUCHI, K. & WHITMORE, W. F. (1971) Epidemiology of cancer of the prostate. *Cancer*, **28**, 344.